# A Role for Electrotonic Coupling Between Cortical Pyramidal Cells

**DOI:** 10.3389/fncom.2019.00033

**Published:** 2019-05-28

**Authors:** Jennifer Crodelle, Douglas Zhou, Gregor Kovačič, David Cai

**Affiliations:** ^1^Courant Institute of Mathematical Sciences, New York University, New York, NY, United States; ^2^School of Mathematical Sciences, MOE-LSC, and Institute of Natural Sciences, Shanghai Jiao Tong University, Shanghai, China; ^3^Department of Mathematical Sciences, Rensselaer Polytechnic Institute, Troy, NY, United States

**Keywords:** electrotonic coupling, synchrony, pyramidal cells, information processing, coincidence detection

## Abstract

Many brain regions communicate information through synchronized network activity. Electrical coupling among the dendrites of interneurons in the cortex has been implicated in forming and sustaining such activity in the cortex. Evidence for the existence of electrical coupling among cortical pyramidal cells, however, has been largely absent. A recent experimental study measured properties of electrical connections between pyramidal cells in the cortex deemed “electrotonic couplings.” These junctions were seen to occur pair-wise, sparsely, and often coexist with electrically-coupled interneurons. Here, we construct a network model to investigate possible roles for these rare, electrotonically-coupled pyramidal-cell pairs. Through simulations, we show that electrical coupling among pyramidal-cell pairs significantly enhances coincidence-detection capabilities and increases network spike-timing precision. Further, a network containing multiple pairs exhibits large variability in its firing pattern, possessing a rich coding structure.

## 1. Introduction

Synchronized neuronal activity in the cortex is essential for information processing underlying many cognitive functions, such as learning, attention, and memory formation (Wang and Buzsaki, 1996; Buzsaki and Draguhn, 2004; Wang et al., 2010). Several sensory regions in the brain, including the olfactory, visual, and auditory cortices, were observed to exhibit synchronized spiking activity when presented with a stimulus (Singer and Gray, 1995; Moore et al., 2001; Haddad et al., 2013).

Experimentalists and computational neuroscientists have shown that, in addition to neuronal communication via chemical synapses, electrical coupling among interneurons, local inhibitory neurons, plays an essential role in generating and maintaining synchronous activity among neurons in the cortex (Gibson et al., 1999; Beierlein et al., 2000; Chow and Kopell, 2000; Tamas et al., 2000; Traub et al., 2001; Amitai et al., 2002; Nomura et al., 2003; Bennett and Zukin, 2004; Ostojic et al., 2009). Such coupling occurs through protein channels called gap junctions that directly connect the interior contents of the cells (Revel and Karnovsky, 1967).

Yet, cortical networks have been shown to exhibit synchronized oscillations typically attributed to gap-junction connectivity even when the gap junctions among interneurons are blocked, suggesting a possible existence of electrical coupling among the excitatory, pyramidal cells (Mercer et al., 2006; Dere and Zlomuzica, 2012). Gap-junction connections between pyramidal cells in the olfactory system of Drosophilia have been shown not only to exist but to be crucial for odor-evoked lateral excitation, a mechanism by which the organism responds to synchronized, odor-specific input (Wilson, 2013). Experiments have shown evidence supporting the existence of electrical connections between the axons of pyramidal cells in the hippocampus (Schmitz et al., 2001), while computational studies have shown that this network of axo-axonal electrically-coupled pyramidal cells can exhibit fast-frequency oscillations and sharp wave-ripple oscillations (Traub et al., 1999, 2012). Another computational study showed that vast networks of electrical coupling among pyramidal cells can produce spatiotemporal patterns of activity in the cortex (Traub et al., 2010).

There has been little experimental evidence, however, that gap-junction coupling exists between pyramidal cells in the mammalian neocortex. Mercer et al. (2006), while measuring gap-junction connectivity in the rat neocortex, discovered one connected pair of pyramidal cells, and later, in 2010, Wang et al. (2010) measured ten pairs of pyramidal cells in the prefrontal and visual cortices of rats and ferrets to have pair-wise electrical coupling deemed “electrotonic” coupling to distinguish it from the typical gap-junction coupling among interneurons.

The electrotonic couplings (ECs) were measured to be rare, occurring only pair-wise between cells that have touching or overlapping soma, with a coupling probability of 5% (Wang et al., 2010), significantly lower than those assumed in previous computational studies and much lower than the 60% coupling probability observed among interneurons separated up to 80 microns (Galarreta and Hestrin, 1999, 2001; Gibson et al., 1999). The junctional conductance of ECs was measured as nearly 25-times higher than that of gap junctions between interneurons. Moreover, action potentials were shown to propagate through the junction, a property that has never been measured for gap-junction-coupled interneurons. The protein that might form this strong, but rare, electrotonic coupling, however, remains unknown, making further experimental investigation difficult and network effects of such a junction undetermined (Connors and Long, 2004; Mercer et al., 2006).

In this work, we address a possible role that EC between *pairs* of pyramidal cells could play in shaping the dynamics of neocortical networks. Through constructing and simulating a realistic model network, we propose that pairs of electrotonically-coupled pyramidal cells, or *electrotonic pairs*, act as sensitive coincidence detectors, eliciting synchronized activity in the network in response to coincident input. Further, we describe how electrotonic pairs could evoke many synchronized network events, with each event more tightly synchronized than those elicited by pyramidal cells without EC. Our results further suggest that multiple electrotonic pairs evoke network events which interact with one another, resulting in a network that can exhibit high variability in its spiking patterns, and thus possesses a rich coding structure.

## 2. Methods

To elucidate the biological function of rare electrotonic pairs in a downstream patch of cortical cells, we set up a multi-layer model network in which the electrotonic pair(s) receive varying levels of synchronized input from an upstream network. Neurons in the downstream network are modeled using the Hodgkin-Huxley (HH) neuronal equations and are organized on a grid, with biologically-relevant connection probabilities and strengths for both synaptic and electric connections and for populations of both inhibitory (interneurons) and excitatory (pyramidal) cells, see below for details. Each neuron, including the electrotonic pair, receives a Poisson background drive modeling incoming spikes from neighboring regions. Since realistic features of network dynamics during sensory stimuli may not be adequately captured by Poisson statistics, we simulate an upstream network using conductance-based Integrate-and-Fire (IAF) neurons. The output of this IAF network is used to mimic sensory information transmitted to the electrotonic pair(s) in the downstream network. The activity and synchrony of the downstream patch are analyzed as a function of the input synchrony, or amount of coincident spikes, received by the electrotonic pair(s).

The following subsections discuss the details of the mathematical models of both the downstream model network and upstream input network. A description of the methods used to quantify synchrony is included in this section as well.

### Mathematical Models

#### The IAF Upstream Model

The dynamics of the *i*th model neuron in the IAF upstream network satisfy the set of equations


$$\begin{array}{ll} C\frac{dv_{i}}{dt} & =-g_{L}\left(v_{i}-\epsilon_{R}\right)-g_{i}^{E}\left(t\right)\left(v_{i}-\epsilon^{E}\right)-g_{i}^{I}\left(t\right)\left(v_{i}-\epsilon^{I}\right),\\ \sigma^{Q}\frac{dg_{i}^{Q}}{dt} & =-g_{i}^{Q}+h_{i}^{Q},\;Q=\left\{E,I\right\},\\ \sigma^{Q}\frac{dh_{i}^{Q}}{dt} & =-h_{i}^{Q}+\underset{j\neq i}{\sum }S_{j}^{Q}\underset{k}{\sum }\delta \left(t-T_{j}^{k}\right)+f^{Q}\underset{k}{\sum }\delta \left(t-T_{i}^{k}\right), \end{array}$$

where *v*_*i*_(*t*) is the voltage, or membrane potential, of the *i*th neuron, *g*_*L*_ is the leak conductance, and $g_{i}^{Q}\left(t\right)$ is the synaptic conductance represented by the characteristic shape of experimentally-observed conductance traces (Johnston and Brown, 1983). The right-hand side of the dynamical equation for $h_{i}^{Q}$ contains two sums: The first sum models incoming spikes from the *j*th presynaptic neuron at times $T_{j}^{k}$ with strength $S_{j}^{Q}$ for both excitatory, *Q* = *E*, and inhibitory, *Q* = *I*, synaptic inputs. The second sum models incoming spikes to neuron *i* at times $T_{i}^{k}$ with strength *f*^*Q*^ that originate from outside the model network. These external spikes are modeled by a Poisson spike train with rate ν. The dynamics of the voltage are such that the incoming spikes from other neurons in the network and inputs from the Poisson spike train increase (excitatory input) or decrease (inhibitory input) the voltage and, in the absence of incoming spikes, the voltage decays exponentially toward the resting potential, ϵ_*R*_. If the voltage is raised such that it reaches a threshold, determined by ϵ_*T*_, the neuron is said to have spiked, the spike time is recorded, and the voltage is set to the reset value, ϵ_*R*_. The procedure for calculating the spike time and efficiently implementing the time-evolution equations can be found in Shelley and Tao (2001). The parameter values used in this model are the dimensional versions of those used in Shelley and Tao (2001) and can be found in Table 1. The IAF model network contains 100 neurons that are all-to-all connected, including 75% excitatory and 25% inhibitory neurons, and results were obtained from simulations of 5 s for each trial.

**Table 1:** Table of parameter values used in the upstream IAF model.

**NEURON PARAMETERS**	
Capacitance, *C*	1 μF/cm^2^
Leak conductance, *g*_*L*_	0.05 mS/cm^2^
Reset potential, ϵ_*R*_	−70 mV
Firing threshold, ϵ_*T*_	−55 mV
Excitatory reversal potential, ϵ^*E*^	0 mV
Inhibitory reversal potential, ϵ^*I*^	−80 mV
Synaptic time constant (exc), σ^*E*^	1.0 ms
Synaptic time constant (inhib), σ^*I*^	4.0 ms
Synaptic strength (exc), *S*^*E*^	0.2 mS/cm^2^
Synaptic strength (inhib), *S*^*I*^	0.4 mS/cm^2^
**NETWORK PARAMETERS**	
External rate, ν	1,000 → 5,000 Hz
External strength *f*^*E*^	11.6 → 12.1 mS/cm^2^
(to excitatory neurons)	
External strength *f*^*I*^	10.0 → 9.2 mS/cm^2^
(to inhibitory neurons)	

*The rate of the Poisson drive ν is described as a range from low to high with corresponding input strengths f^Q^ chosen to maintain an average constant firing rate of the neurons with varying levels of synchrony*.

#### The HH Downstream Model

The neurons in the downstream network are modeled using a modified version of the HH equations, with an additional ohmic term describing the current through the junctions, for both interneurons and pyramidal-cell pairs. Previous work has modeled gap junctions among interneurons using IAF neurons with the inclusion of an instantaneous jump in the voltage of the post-junctional cell in response to spiking in the pre-junctional cell (Lewis and Rinzel, 2003) or by inserting characteristic action potentials using a spiking kernel (Chow and Kopell, 2000). Here, we use the more complex HH model because EC has a very high conductance, strongly coupling the membrane potential of the pair of neurons at all times, including during an action potential. As was shown experimentally by Wang et al. (2010), EC can propagate full action potentials, producing changes in the voltage of the post-junctional cell that depend on the shape and size of the pre-junctional action potential.

The voltage of the *i*th downstream neuron is described by the equation


$$\begin{array}{l} C\frac{dv_{i}}{dt}=-g_{L}\left(v_{i}-v_{R}\right)-{ḡ}_{Na}m^{3}h\left(v_{i}-v_{Na}\right)-{ḡ}_{K}n^{4}\left(v_{i}-v_{K}\right)\\ \;-g_{C}\underset{j}{\sum }\left(v_{i}-v_{j}\right)-G_{i}^{E}\left(t\right)\left(v_{i}-v^{E}\right)-G_{i}^{I}\left(t\right)\left(v_{i}-v^{I}\right), \end{array}$$

where ḡ_*Na*_ and ḡ_*K*_ are the maximal sodium and potassium conductances, and *v*_*Na*_ and *v*_*K*_ are the sodium and potassium reversal potentials, respectively. In addition, activation and inactivation parameters, *m*, *h*, and *n*, modeling the opening and closing of the ion channels, exhibit dynamics described by the differential equation


$$\begin{array}{ll} \frac{dz}{dt} & =\alpha_{z}\left(v\right)\left(1-z\right)-\beta_{z}\left(v\right)z,\;z=m,n,\;h, \end{array}$$

with each rate variable described by a set of voltage-dependent equations


$$\begin{array}{ll} \alpha_{m}\left(v\right) & =\frac{-0.32\left(v-v_{T}-13\right)}{\exp\left[-\left(v-v_{T}-13\right)/4\right]-1}\\ \beta_{m}\left(v\right) & =\frac{0.28\left(v-v_{T}-40\right)}{\exp\left[\left(v-v_{T}-40\right)/5\right]-1}\\ \alpha_{h}\left(v\right) & =0.128\exp\left[-\left(v-v_{T}-17\right)/18\right]\\ \beta_{h}\left(v\right) & =\frac{4}{1+\exp\left[-\left(v-v_{T}-40\right)/5\right]}\\ \alpha_{n}\left(v\right) & =\frac{-0.032\left(v-v_{T}-15\right)}{\exp\left[-\left(v-v_{T}-15\right)/5\right]-1}\\ \beta_{n}\left(v\right) & =0.5\exp\left[-\left(v-v_{T}-10\right)/40\right], \end{array}$$

as determined by Pospischil et al. (2008) using fitting techniques to match both fast-spiking (interneurons) and regular-spiking (pyramidal) cells' activity in the cortex.

The synaptic conductance is described using fourth-order kinetics as was done by Sun et al. (2010) by the equations


$$\begin{array}{rc} \frac{d}{dt}G_{i}^{Q}\left(t\right) & =-\frac{G_{i}^{Q}\left(t\right)}{\sigma_{r}^{Q}}+G_{i1}^{Q}\left(t\right)\\ \frac{d}{dt}G_{i1}^{Q}\left(t\right) & =-\frac{G_{i1}^{Q}\left(t\right)}{\sigma_{r}^{Q}}+G_{i2}^{Q}\left(t\right),\\ \frac{d}{dt}G_{i2}^{Q}\left(t\right) & =-\frac{G_{i2}^{Q}\left(t\right)}{\sigma_{r}^{Q}}+G_{i3}^{Q}\left(t\right),\\ \frac{d}{dt}G_{i3}^{Q}\left(t\right) & =-\frac{G_{i3}^{Q}\left(t\right)}{\sigma_{r}^{Q}}+G_{i4}^{Q}\left(t\right), \end{array}$$

(1) $$\begin{array}{l} \frac{d}{dt}G_{i4}^{Q}\left(t\right)=-\frac{G_{i4}^{Q}\left(t\right)}{\sigma_{r}^{Q}}+\underset{j\neq i}{\sum }S_{j}^{Q}h\left(v_{j}^{\text{pre}}\right)+\underset{k}{\sum }f_{\text{back}}\delta \left(t-T^{k}\right)\\ \;\\ +\underset{\ell }{\sum }f_{\text{sens}}\delta \left(t-T^{\ell }\right), \end{array}$$

where


$$\begin{array}{l} h\left(v\right)=\frac{1}{1+\text{exp}\left(-\left(v-20\right)/2\right)}. \end{array}$$

Note that by using a smooth function *h*(*v*), the synaptic interactions are no longer event-driven, but instead depend on the value of the voltage of the presynaptic neuron. The second term in Equation (1) sums over all pre-synaptic neurons *j*, which are chosen randomly with probabilities as described in Table 2. The parameter set used in this work, chosen through matching with voltage-clamp experimental data for electrotonically-coupled pyramidal cells (Wang et al., 2010) and gap-junction connected fast-spiking neurons (Galarreta and Hestrin, 1999), can be found in Table 2. The networks consists of 75% excitatory (pyramidal) cells and 25% inhibitory cells as is understood to occur in the neocortex of mammals (Beaulieu, 1993) and has been done in previous realistic modeling studies (Cai et al., 2005).

**Table 2:** Table of parameter values used in the downstream HH network model.

	**FS cells**	**Pyramidal Cells**
**NEURON PARAMETERS**			
Capacitance, *C* (μF/cm^2^)	1	1
Leak conductance, *g*_*L*_ (mS/cm^2^)	0.1	0.025
Reset potential, *v*_*R*_ (mV)	−70	−70
Maximal *Na* conductance, ḡ_*Na*_ (mS/cm^2^)	30	55
Maximal *K* conductance, ḡ_*K*_ (mS/cm^2^)	5	3
Threshold, *v*_*T*_ (mV)	−58	−45
*Na* reversal potential, *v*_*Na*_ (mV)	30	55
*K* reversal potential, *v*_*K*_ (mV)	−90	−80
Excitatory reversal potential, *v*^*E*^ (mV)	0	0
Inhibitory reversal potential, *v*^*I*^ (mV)	−80	−80
Synaptic time constant, $\sigma_{r}^{E}$ (ms)	0.4	0.4
Synaptic time constant, $\sigma_{r}^{I}$ (ms)	1.0	1.0
Synaptic strength (exc), *S*^*E*^ (mS/cm^2^)	0.4	0.4
Synaptic strength (inhib), *S*^*I*^ (mS/cm^2^)	0.4	0.2
Gap-junction conductance, *g*_*C*_ (mS/cm^2^)	0.012	0.08
**NETWORK PARAMETERS**		
Connection probability, *P*^*E*^ (%)	25	30
(from excitatory neurons)		
Connection probability, *P*^*I*^ (%)	50	20
(from inhibitory neurons)		
Sensory external drive, ν_sens_ (Hz)	100	100
Strength of sensory drive, *f*_sens_ (mS/cm^2^ )	10.0	3.5
Background external drive, ν_back_ (Hz)	5,000	5,000
Strength of background drive, *f*_back_ (mS/cm^2^)	0.44	0.2

*Some parameters were chosen from the experimental studies as performed by Pospischil et al. (2008), others were chosen through matching voltage-clamp experimental data for electrotonically-coupled pyramidal cells (Wang et al., 2010) and gap-junction connected fast-spiking neurons (Galarreta and Hestrin, 1999)*.

### Network Organization

We simulate 400 HH neurons organized on a 20 × 20 grid with their probability of connectivity decaying exponentially with distance, capturing the exponential decrease in synaptic connectivity with cell distance as was done by McLaughlin et al. (2000). Then, the probability of connecting to a neuron on the grid a distance *l* in the horizontal direction and *m* in the vertical direction is given by


$$\begin{array}{l} P^{Q}\left(l,m,r\right)=P^{Q}\text{exp}\left[-\frac{{\left(\sqrt{l^{2}+m^{2}}-1\right)}^{2}}{2r}\right], \end{array}$$

where *r* = 20. The external drive to both networks is drawn from a Poisson process with rate ν and strength *f*^*Q*^, for the upstream, IAF network, and ν_back_ and *f*_back_ for the background drive to the downstream, HH network. This models input from neurons external to the patch of neurons that is explicitly being modeled. In addition, a fraction of the downstream population (20% of inhibitory cells and 30% of excitatory cells) receives an additional external drive modeling input from neurons external to the network that are firing in response to a sensory stimulus, mimicking the increased activity of a subset of neurons during a sensory experience. This additional sensory drive is modeled using a Poisson process with rate ν_sens_ and strength *f*_sens_. Note that all figures were generated using 30 realizations of simulation runs for 5 s of simulated time in each trial.

**Figure 1:**
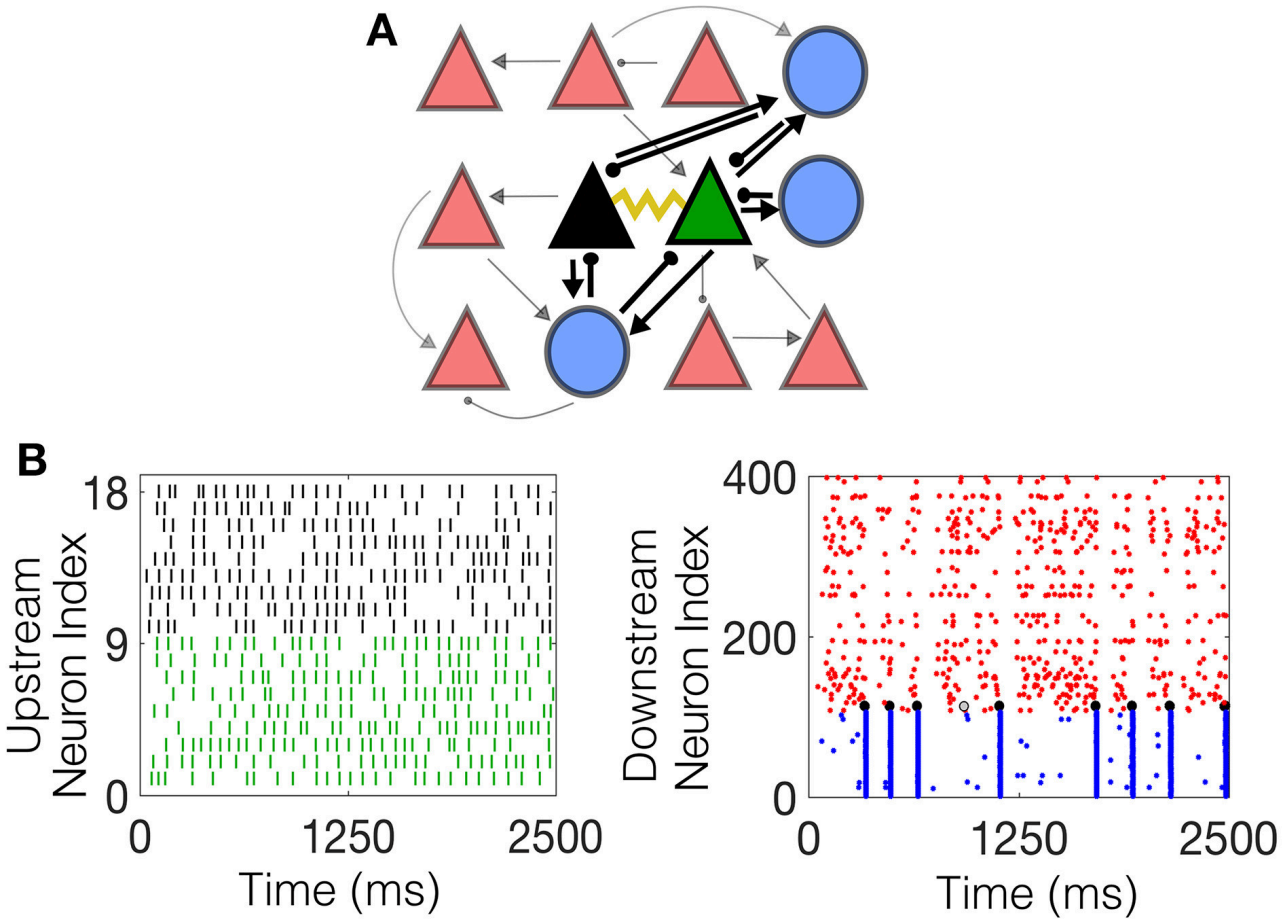
The schematic downstream network architecture, as well as example dynamics of both the upstream IAF input network and the resulting downstream HH network. **(A)** The schematic downstream network. Blue circles denote interneurons, red triangles denote pyramidal cells, and the black and green triangles represent the NDEP. Black lines connecting the cells represent synaptic connections, with the ball at the end representing inhibition, and the arrow excitation. The weight of each line represents the strength of the connection. The yellow curve represents EC between the NDEP. **(B)** (Left) Example raster plot of the incoming spikes from the IAF network received by each neuron in the NDEP. The colors indicate the neuron in the NDEP, see **(A)**, that receives each set of spikes. (Right) Example raster plot of the downstream network resulting from the upstream input, where red dots indicate times at which excitatory neurons fire, and blue dots indicate firing times of the inhibitory neurons. The black dots denote times at which the NDEP fires together (within 5 ms of each other) and the gray dot indicates that just one neuron in the NDEP fired at that time.

The inhibitory neurons are coupled with a gap junction with a coupling probability of 60% and neighboring excitatory pyramidal cells are coupled with a probability of 5%, reflecting experimental findings. One pair of electrotonically-coupled neurons, denoted the *network-driven electrotonic pair (NDEP)*, is selected from the set of all electrotonically-coupled pairs in the network to be driven by a subset of neurons from the IAF network; the NDEP is denoted by the green and black triangles in Figure 1A. The IAF network is simulated prior to the HH model and input spikes are received by each neuron in the NDEP with strength *f*_sens_. The input neurons are chosen randomly from the IAF network such that ~9–10 IAF neurons project to one neuron in the NDEP (the average firing rate received by each neuron in the NDEP is ~100 Hz), with no IAF neuron projecting to both cells in the NDEP. The spike times of these input IAF neurons, shown in Figure 1B together with an example of the resulting dynamics of the downstream network in Figure 1C, are modeled as received by the NDEP in the same way as external spikes to network neurons (i.e., through a delta function in the *G*_4_ variable at the input neuron spike times).

We postulate that synapses between the NDEP and the inhibitory cells are stronger than the average synapse between other cells in the network. The NDEP excites the gap-junction connected inhibitory cells to fire synchronous action potentials, which allows the synapses between the NDEP and the interneuron population to be preferentially enhanced through synaptic plasticity and classic learning rules (Feldman, 2012). As a result, we include a 10-fold increase in the synaptic strength from the NDEP to the interneurons and a reciprocal 3-fold increase in synaptic strength from the interneurons back to the NDEP, see Table 2 for normal synaptic strength values. This structure allows for a clear understanding of the effect of EC between a pair of pyramidal cells on network dynamics as discussed below.

The following sections describe the methods used to quantify the synchrony of the upstream IAF neurons' spike times, as well as the synchrony exhibited by the downstream network.

### Input Synchrony

**Figure 2:**
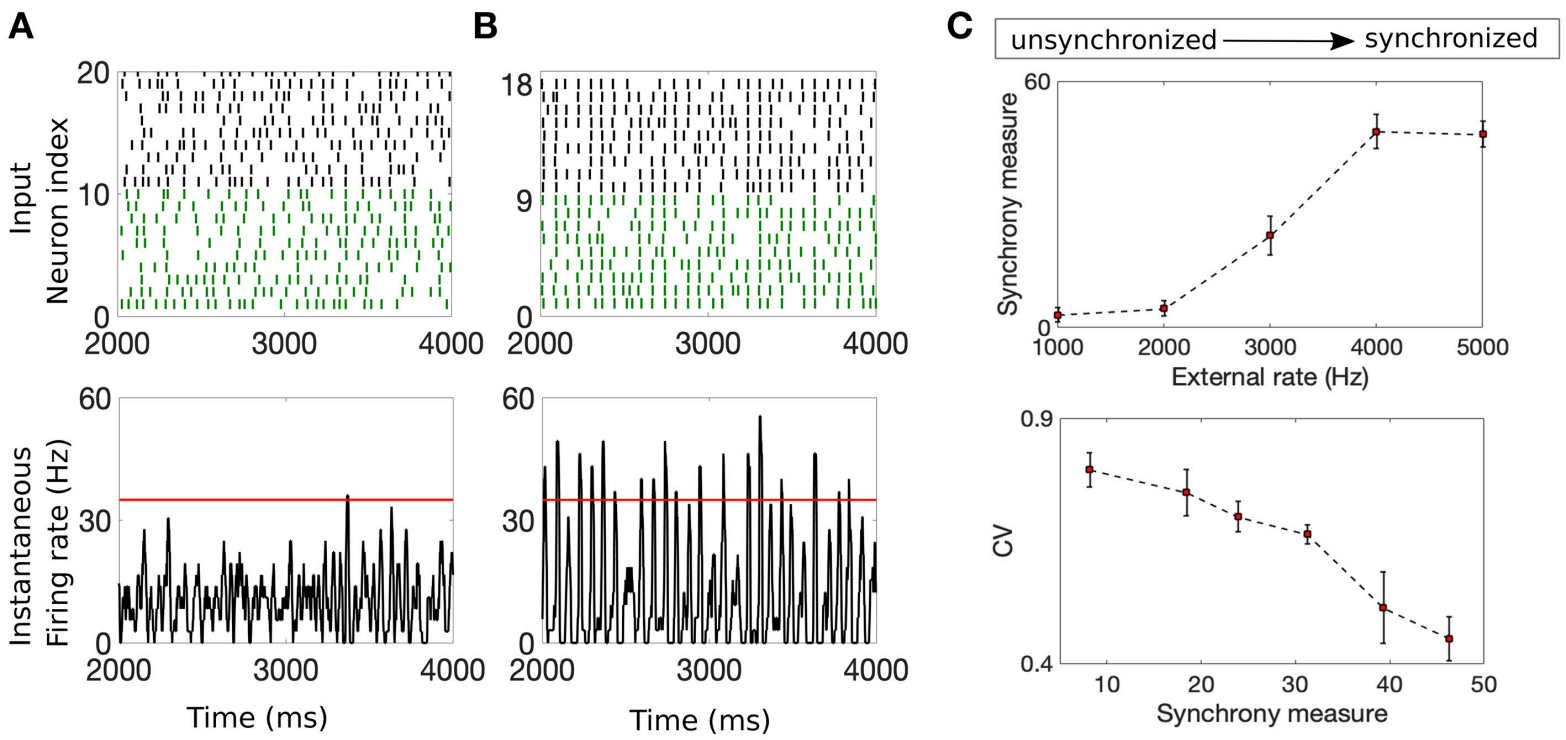
Example networks with two different input synchrony measures. **(A,B)** (Top) Raster plots of the incoming spike times from the IAF upstream network and (bottom) plots of the smoothed instantaneous firing rate in the unsynchronized case **(A)** and the synchronized case **(B)**. The red line denotes the threshold value of 35 Hz. The input synchrony value, or number of times the instantaneous firing rate crosses the threshold, for these two cases are 1 for the input network in **(A)** and 18 for the input network in **(B)**. **(C)** (Top) The input synchrony measure as a function of the rate of the background drive, ν (from unsynchronized to synchronized network activity) to the IAF network. (Bottom) The coefficient of variation (CV) of the interspike interval of the IAF neurons as a function of the input synchrony value. Note that values close to 1 indicate spiking statistics similar to Poisson, while values close to 0 indicate spiking statistics more regular than Poisson. The strength of the external drive, *f*^*E*^ and *f*^*I*^, are adjusted with changes in the rate ν such that the product *f*^*Q*^ν is a constant, see Table 1 for values.

Input synchrony is a measure of the amount of synchronous input received by each neuron in the NDEP from the IAF upstream network. We determine it as follows. First, the instantaneous firing rate in each trial is calculated by binning time into 2 ms-sized bins and counting the number of spikes from the IAF network that occur in each bin (Shinomoto, 2010). This count is converted into an average firing rate by dividing by the total number of neurons and the length of the time bin. Then, a smooth curve is created using a moving-window average with a smoothing width of 5 ms. We determine the value of the input synchrony by counting the total number of times in all trials that the smoothed instantaneous firing rate crosses a threshold of 35 Hz, i.e., the number of times the spikes from many neurons in the network fall within a short window of time. The threshold is chosen as the maximum of the smoothed instantaneous firing rate for a completely asynchronous network, yielding an input-synchrony value close to zero for the asynchronous case, and guaranteeing that the measure will increase with an increase in synchrony. Note that this threshold is kept constant throughout all simulations and the results are not sensitive to moderate changes in this threshold. In this study, we adjust the input synchrony by varying the statistical properties of the Poisson external drive to the IAF network from fluctuation-dominated to mean-dominated, see Figure 2 for example networks with different input synchrony values. The input synchrony values are computed per trial. Clearly, the input synchrony measure correlates well with the coefficient of variation (CV) of the interspike interval, a measure for the regularity of the spiking statistics, see Figure 2C. However, we note that CV values give no indication of synchrony, or alignment of spikes in time, so we use the synchrony measure throughout the remainder of this work.

### van Rossum Distance

The van Rossum method determines a distance, in time, between two pairs of spike trains by adding exponential tails to each spike time for each neuron and integrating the square of the difference between these exponential-tailed spike times (van Rossum, 2001). It is calculated as follows: First, the spike times of two neurons, *x* and *y*, are denoted as $\left\{t_{i}^{x}\right\}$ and $\left\{t_{j}^{y}\right\}$, respectively. Then, exponential tails with time constant τ_*c*_ are added to each spike time, $t_{i}^{x}$, to create a smeared-spike function, *x*(*t*), with the following equation:


$$\begin{array}{l} x\left(t\right)=\underset{i}{\sum }H\left(t-t_{i}^{x}\right)e^{-\left(t-t_{i}^{x}\right)/\tau_{c}}, \end{array}$$

where *H*(*t*) is the Heaviside function. The smeared-spike function for the second neuron, *y*(*t*), is defined analogously. Finally, the van Rossum distance between the spike trains is defined as the integral of the square of the difference,


$$\begin{array}{l} D^{2}\left(x,y\right)=\frac{1}{\tau_{c}}\int_{0}^{\infty }{\left[x\left(t\right)-y\left(t\right)\right]}^{2}\;dt. \end{array}$$

The result is that *D*^2^ is large for spike trains that do not align in time (are unsynchronized), and small for those that do (are synchronized). In this work, the time constant for the exponential tail, τ_*c*_, is set to 5 ms as was done in previous modeling studies (Barranca et al., 2014) and is recommended for detecting synchrony on short time scales. Note that large values of τ_*C*_ would yield an approximate difference in the total number of spikes elicited by the two neurons instead of the spike times.

### Spike-Difference (SD) Method

We developed the spike-difference (SD) method to detect small changes in synchrony. Due to the gap-junction connectivity among the interneurons, the network exhibits synchrony for nearly all types of external drive, as well as in the presence or absence of a rare number of electrotonic pairs. The intuition is as follows: Once the network is sufficiently excited to induce firing, the interneurons fire synchronously due to their extensive gap-junction connections, causing a network synchronous event (NSE). Specifically, at the time of the NSE, the excitatory neurons are firing sufficiently to excite the interneuron population to threshold, creating a wave of inhibition that suppresses the activity of the entire population for a brief period of time succeeding the NSE. This can be seen in Figure 1B (right) by noting the lack of network spikes immediately after each NSE, where the NSE is indicated by the wave of inhibitory neurons spiking within a short window of time.

**Figure 3:**
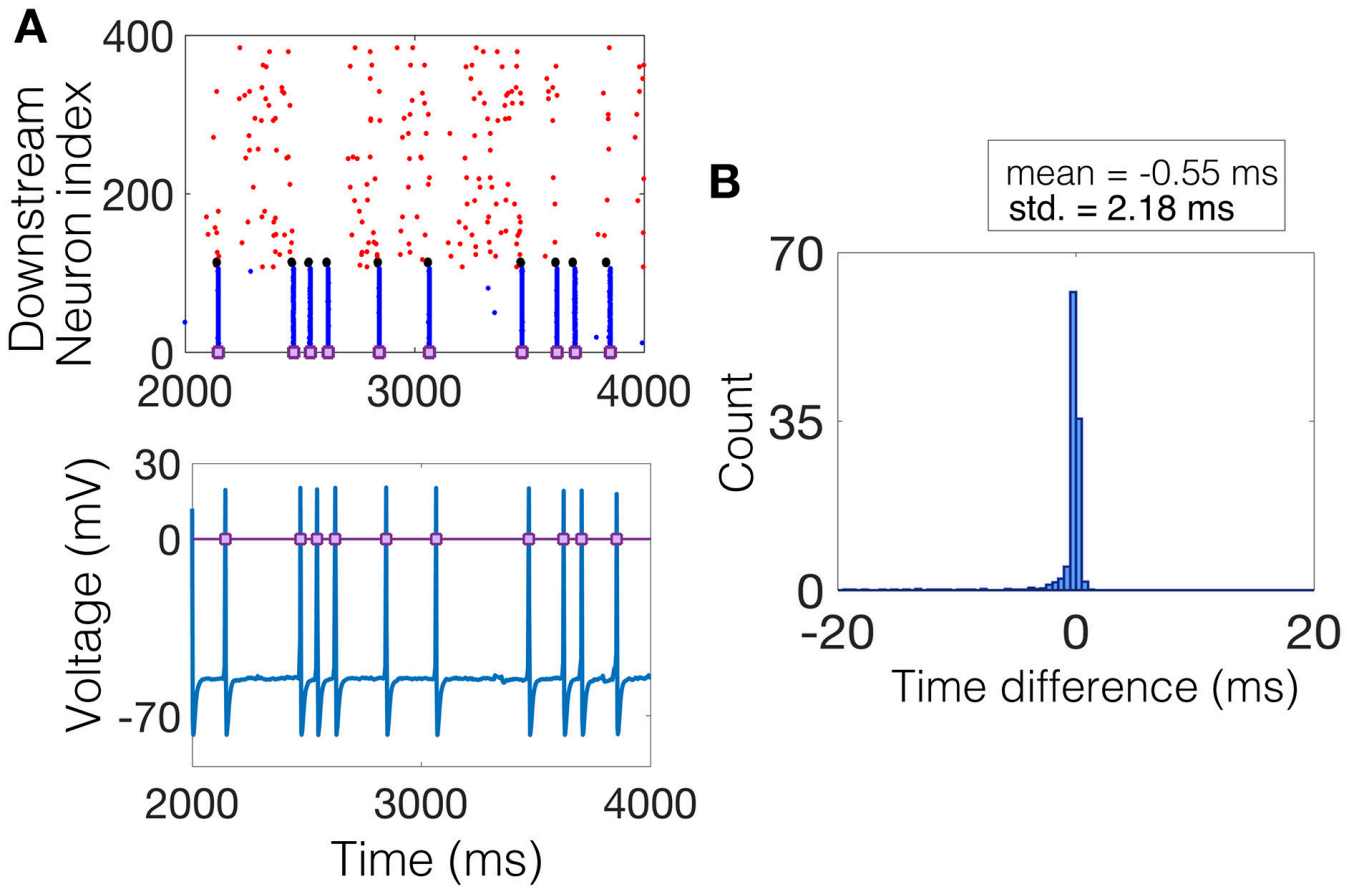
Computing the SD measure. **(A)** (Top) Example raster plot where the spike times of the excitatory neurons are shown in red, the spike times of the inhibitory neurons are shown in blue, and the spike times of the NDEP are shown black. The purple squares denote times at which an NSE occurred. (Bottom) The corresponding average voltage of the inhibitory neurons, where the purple squares denote the NSE times determined by the times at which the average voltage crosses a threshold of 0 mV, marked by the purple line. **(B)** The histogram of time-differences from each NSE to the spike times of all neurons that fall within 20 ms of the NSE. The SD measure is taken to be the standard deviation of this histogram, a value of 2.18 in this example, as shown in bold in the legend.

The SD method measures, for each NSE, the amount of synchrony exhibited by all neurons that participate in the event. The procedure is as follows. The time of the NSE is found by recording the times at which the magnitude of the average voltage of the interneuron population increases by 70 mV. This magnitude is kept constant throughout the simulations and the results do not change with moderate changes in this choice of magnitude, see Figure 3A for a raster plot of an example realization of the downstream network and the corresponding average voltage, with the threshold plotted as a purple line. Then, within a window of time around the NSE time, e.g., ±20 ms, the time-difference between each spike and the NSE time is measured for each neuron in each trial that spiked within this time window. The result is a distribution of time-differences in all trials, centered near 0 ms, the width, i.e., standard deviation, of which determines the average amount of synchrony the network exhibited during each NSE, see Figure 3B.

## 3. Results

### EC Enhances Synchrony Between a Pyramidal-Cell Pair

**Figure 4:**
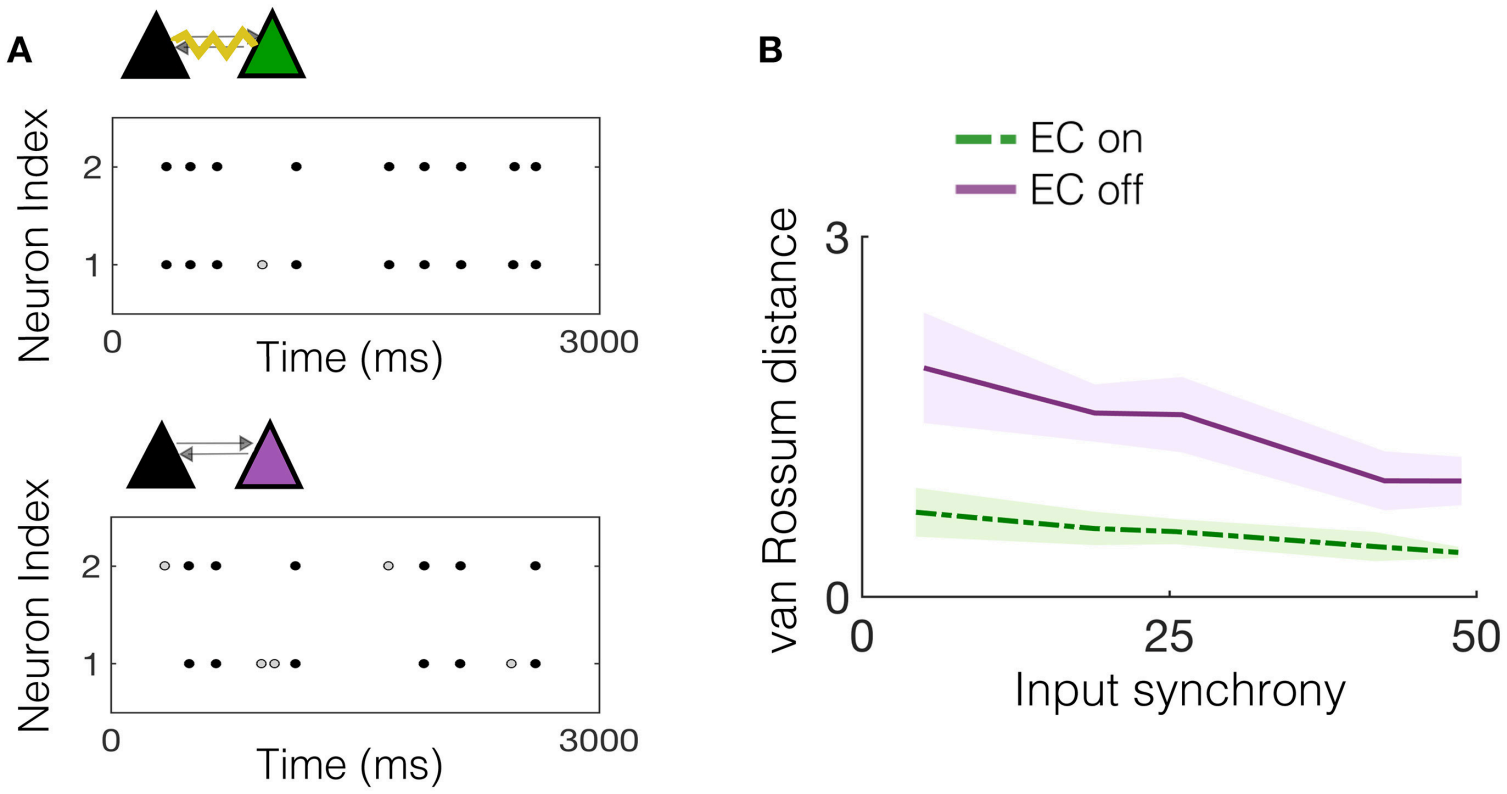
Experimentally-measured EC conductance induces synchronized spiking between neurons in the NDEP. **(A)** (Top) An example realization of the spike times of the NDEP for an input synchrony value of 30 for the case with EC turned on (non-zero conductance) and (bottom) spike times of the same NDEP, but with EC turned off (zero conductance). The gray filled circles denote times at which only one neuron in the NDEP fires, whereas the black circles show times at which both neurons in the NDEP fire within 5 ms of one another. **(B)** Mean (line) and standard deviation (shading) of the van Rossum distance between the spike times of the neurons in the NDEP in response to several values of the input synchrony.

We begin by considering the effect of EC between one pair of neurons that is embedded in the downstream network and receives input from a subset of upstream IAF neurons exhibiting varying amounts of synchrony. First, we note that the two pyramidal cells in the NDEP have synchronized activity due to their EC, which strongly couples the voltages of the two neurons, resulting in almost identical spike trains. As an example, Figure 4A shows the spike times for the NDEP with experimentally-measured electrotonic conductance, compared with the spike times of the same two neurons with EC turned off, i.e., the junctional conductance is set to zero. Notice that the spike trains of the NDEP with EC turned on are almost identical, firing almost every spike together, whereas the synchronized firing between neurons in the NDEP is largely reduced with EC turned off.

We quantify the amount of synchrony exhibited by the spike trains in both cases (EC on and off) using the van Rossum distance, see Figure 4B. As expected from the near-identical spike trains, the distance between the spike times for the NDEP with EC turned on is significantly smaller than for the NDEP with EC turned off, for all input-synchrony values. Importantly, note that the structure and parameter values of the network with EC turned off are identical to the case with EC turned on, with the only difference between the two cases residing in the conductance of the EC between the two neurons in the one NDEP. The implication of this significant decrease is that downstream neurons will receive two synchronized excitatory spikes instead of two asynchronous ones, suggesting a role for the NDEP in organizing network activity around its own synchronized firing pattern.

### One NDEP Increases Precision of Network Spike Timing

**Figure 5:**
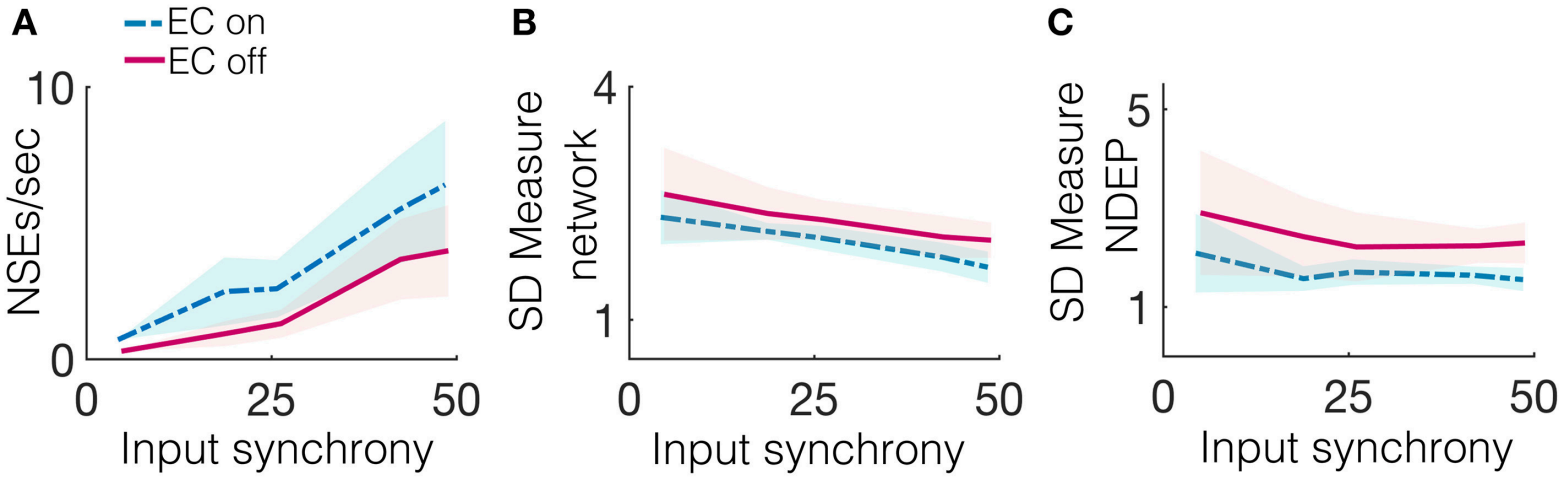
Several synchrony measures for a network containing one NDEP, comparing the case of EC on and off. The synchrony measures are: **(A)** The number of NSEs per second, **(B)** The SD measure using the spike times of all neurons in the network (excluding the NDEP), and **(C)** The SD measure using the spike times of just the two neurons in the NDEP, for the case when EC is turned on (blue, dash-dotted) and off (pink, solid) over all input-synchrony values.

Due to the gap junctions that ubiquitously couple the network of inhibitory neurons, input from the excitatory population (whether the input is from neurons connected by a chemical synapse or by EC) creates synchronized firing events called NSEs (as described previously in the Methods section). The synchronization of neuronal spiking is often observed in response to sensory stimuli, with the timing of these synchronized events thought to be important for downstream communication (Azouz and Gray, 2003). The number of NSEs, which one can interpret as a network firing rate, is a relevant quantity of interest because it can also provide a measure for the amount of information transmitted from the model patch to a further downstream network. Figure 5A shows that the network containing an NDEP with EC turned on generates a greater number of NSEs than a network containing that same NDEP with EC turned off. Note that the NDEP with EC turned off still has the increased synaptic connection to and from the inhibitory network population, but that these two neurons exhibit significantly less synchronized spiking than two neurons with EC turned on, and so the inhibitory population does not often receive enough synchronized spikes to elicit an NSE in response to the firing of the NDEP. This means that the NDEP with EC turned off requires more input synchrony to elicit the necessary synchronized spiking to excite the inhibitory population and create an NSE. The NDEP with EC turned on, however, requires a less synchronous input to generate the same number of NSEs than an NDEP with EC turned off and thus we observe that the non-zero EC creates an NDEP that reduces temporal jittering of spikes and enhances the associations between spike events.

Firing rate is not the only method by which neuronal networks communicate and encode information. Rather, some neurons respond maximally to changes in spike timing on short timescales, i.e., tight synchrony (Singer, 1993). Figure 5B shows the SD measure using all neurons in the network (a measure of the tightness of the synchrony of each NSE, see the Methods section for the algorithm to compute the SD measure) to show that the NDEP with non-zero conductance creates NSEs that are tightly synchronized. The network in which the NDEP has zero conductance (EC is turned off) still generates NSEs; however, these events are less tightly synchronized than when EC is turned on, resulting in less communication to downstream areas. We also reinforce the result from the van Rossum distance (that the two neurons in the NDEP are tightly synchronized, recall Figure 4B), and validate the SD measure, by calculating the SD measure using only the spike times of the NDEP, see Figure 5C. Note that trend follows similarly as in the van Rossum distance, and we observe that the tight synchrony induced by the NDEP may aid in coincidence detection achieved by neurons in a downstream network.

**Figure 6:**
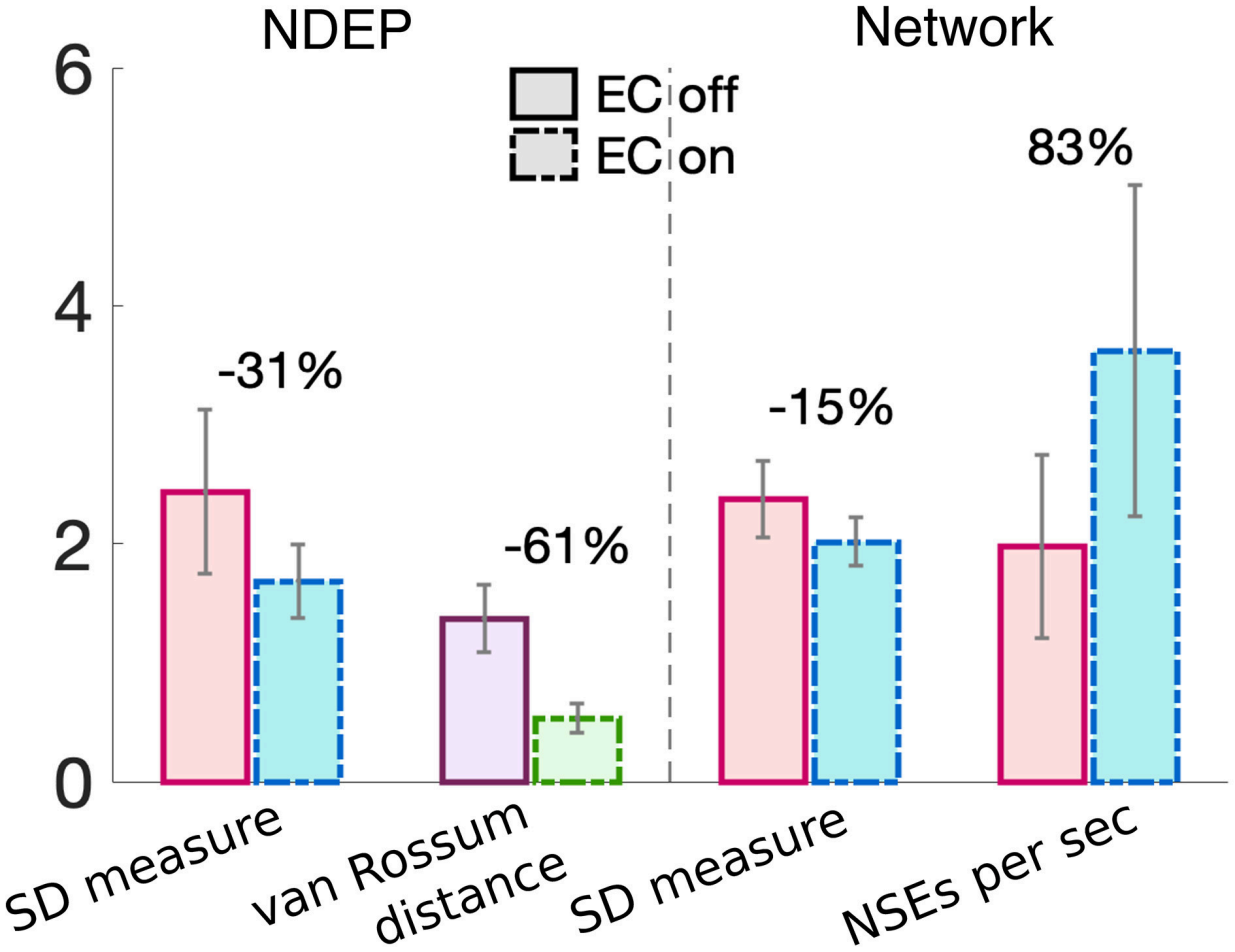
Summary of the changes in each synchrony measure from turning EC on (dash-dotted bars) to EC off (solid bars) for just the neurons in the NDEP (left of the dashed line) and all neurons in the network (right of the dashed line). Percent change is given on top of each set of bars and was calculated from the case with EC turned off to EC turned on. Gray lines indicate standard deviation.

These results together demonstrate that the NDEP with EC turned on elicits more NSEs than this same NDEP with EC turned off and, further, that these NSEs are composed of spike times that are closer together in time, i.e., more synchronized. However, notice that, in all three measures, EC turned on or off does not influence the behavior of the network as the input synchrony changes (see Figure 5 and notice that the lines appear nearly parallel). Therefore, in Figure 6, we quantify by averaging over all input-synchrony values the change in the van Rossum distance between the spike times of the neurons in the NDEP (Figure 4B), the synchrony (SD measure) of the neurons in the NDEP for each NSE (Figure 5C), the total number of NSEs (Figure 5A), and the network synchrony (SD measure) of each NSE (Figure 5B) for the case when EC is turned on and off. These results combine to support the hypothesis that one NDEP with non-zero conductance can act to reduce the noise in an incoming stimulus (i.e., create a more synchronous event in response to less synchronous input) and increase the precision of network spike timing (i.e., tighten these synchronous events).

### Two NDEPs Enhance Discriminability of Incoming Stimuli

Since Wang et al. (2010) identified several pairs of electrotonically-coupled pyramidal cells in the cortex, we next analyze a network in which an additional NDEP receives input from an upstream IAF network. Note that this additional NDEP is chosen randomly from the set of all electrotonic pairs in the network and has the same increase in synaptic strength to and from the interneuron network as in the one-NDEP case (see Methods section and Figure 1A). In addition, we note that the input to each NDEP can originate from disjoint sets of neurons in the *same* IAF network (SIAF) or from sets of neurons in two *different* IAF networks (DIAF) that have identical parameters (statistics), but differ in their incoming spike times (realizations of Poisson spike train). We investigate the dynamics of the downstream network in response to both types of IAF upstream input.

**Figure 7:**
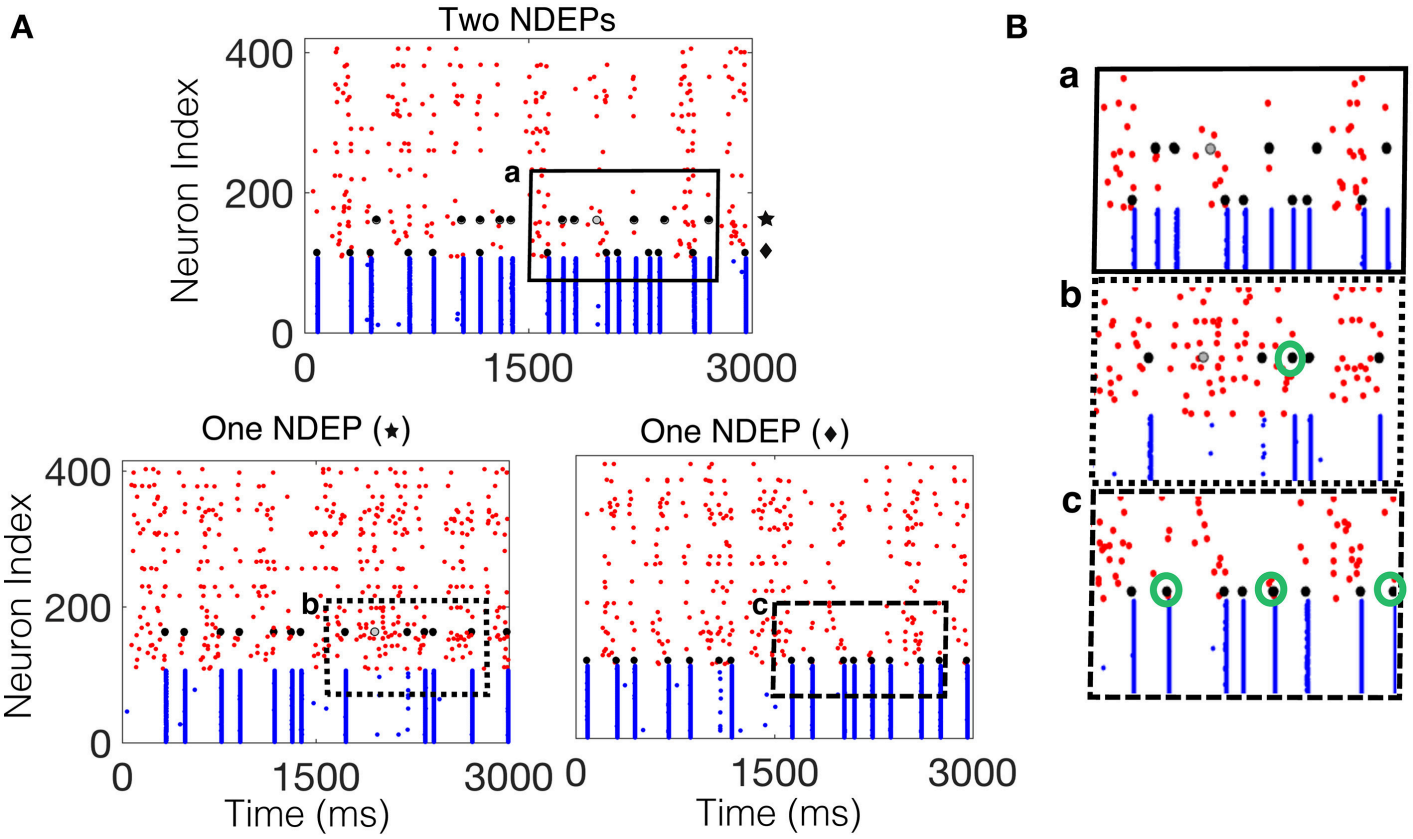
Spiking interactions of two NDEPs in a network receiving DIAF input. **(A)** (Top) Raster plot of the downstream network with two NDEPs receiving DIAF input, with the symbol “star” and “diamond” labeling each NDEP. (Bottom) Raster plots of the downstream network with one NDEP chosen as the pair labeled with the star (left) and with the diamond (right). **(B)** Example of several interactions that occur in the network containing two NDEPs (a, top panel) as demonstrated by comparing to each one-NDEP network (b,c bottom panels). Lettering coordinates with the portion of each raster plot that is being plotted. Green circles indicate those spikes that were deleted in the network in which there are two NDEPs, as compared to their respective raster plots with one NDEP.

For the case in which two NDEPs receive DIAF input, we observe that the two NDEPs compete with one another through the inhibition evoked by each NSE, resulting in network behavior that is more variable than the case in which just one NDEP is present. Figure 7 demonstrates this competition by comparing the network containing two NDEPs (Figure 7A, top) to the network in which just one pair is chosen as the NDEP (Figure 7A, bottom), with the chosen pair indicated by the symbol “star” or “diamond.” In comparing these networks, we determine how the behavior of each NDEP changes when an additional NDEP is present in the network. Notice that, in the network containing two NDEPs, there are several cases in which one NDEP fires, induces an NSE, and the wave of activity from the NSE inhibits the second NDEP from firing. This effect is shown in Figure 7B by the green circles in the bottom two panels, indicating those spikes that have been deleted by the competition between the NDEPs in the network receiving DIAF input.

**Figure 8:**
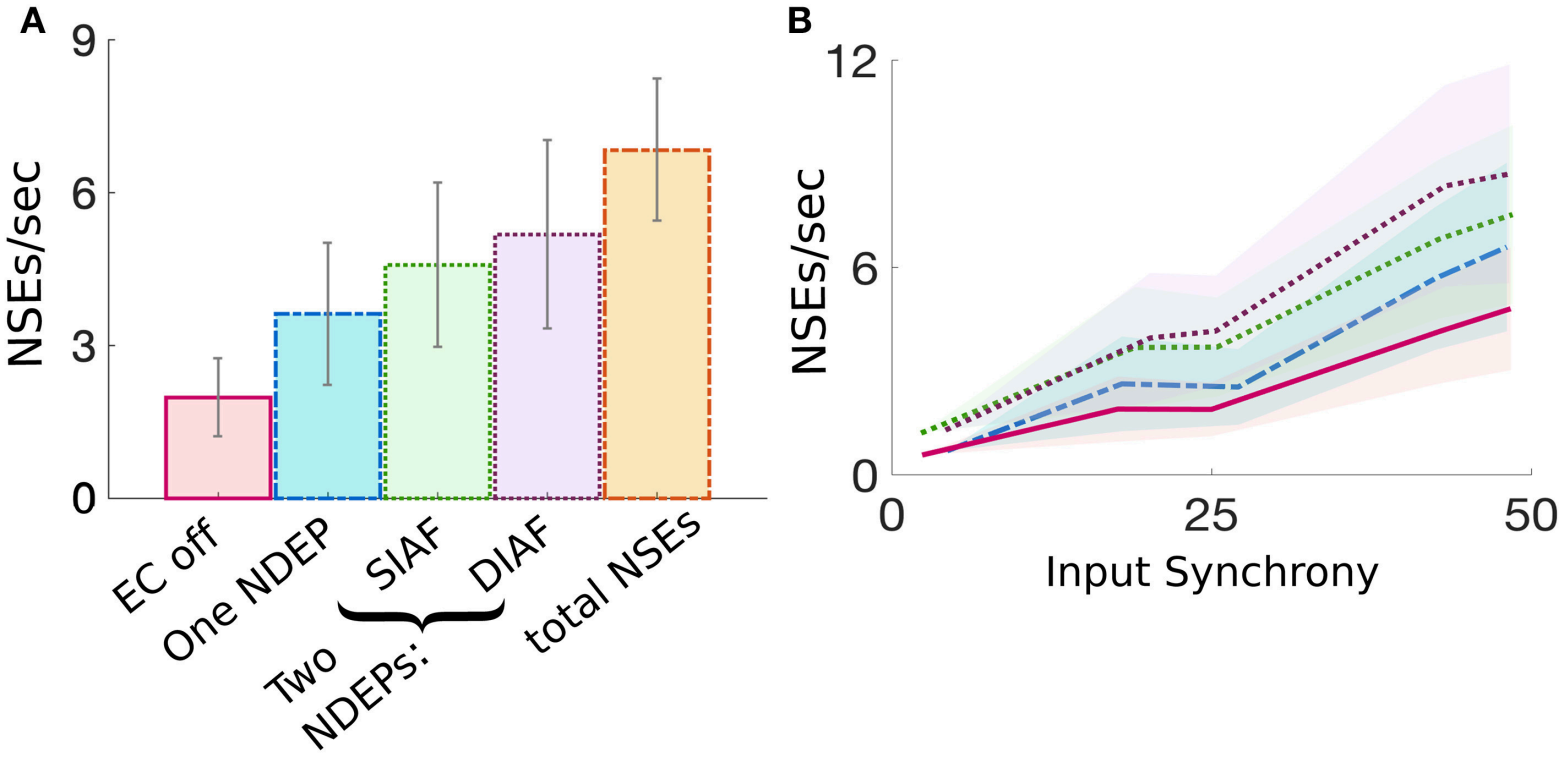
Comparison of the measure of NSEs per second for all network regimes. **(A)** Average number of NSEs per second over all input synchrony values for each regime, with the addition of a regime entitled “total NSE's” in which no interactions between pairs is assumed and the number of NSEs for the one-NDEP with EC on is doubled. Gray lines indicate standard deviation. **(B)** Number of NSEs per second plotted over different input synchrony values for each regime. EC off (pink solid), one NDEP (blue dot-dashed), two NDEPs receiving SIAF input (green dotted), two NDEPs receiving DIAF input (purple dotted), hypothetical two-NDEP network in which there is no competition (orange dash-dotted).

The dynamics of a network containing two NDEPs differs from that containing one NDEP in several ways. First, we investigate how the average number of NSEs varies in each regime (i.e., EC turned off, one NDEP, two NDEPs receiving SIAF input, and two NDEPs receiving DIAF input). Figure 8A shows that a network containing two NDEPs generates a larger number of NSEs than a network with no electrotonic coupling (EC off) and a network with only one NDEP (EC on). Additionally, note that the number of NSEs generated by the two-NDEP network receiving DIAF input (purple bar) is smaller than the number generated by a hypothetical network in which there are no interactions between NDEPs (orange bar), calculated by doubling the number of NSEs in the one-NDEP case.

Figure 8A also demonstrates that the average number of NSEs generated by the two-NDEP network receiving DIAF input is larger than the number that is generated by the network in which the two NDEPs receive SIAF input (green bar). Figure 8B shows that this difference in the number of NSEs generated by each two-NDEP network becomes prominent when the input synchrony values become large (>25). The divergence of these two curves can be explained as follows. When two NDEPs are driven by the same IAF network, the spike times received by each NDEP become closer together as the input network synchronizes. As a result, the two NDEPs receive spikes at sufficiently close times so that their spike timing will be sufficiently close to elicit one NSE in response. In contrast, when two NDEPs are driven by different IAF networks, the spike times of the input neurons are sufficiently far apart so that their spike timing will also be sufficiently far apart to potentially elicit two distinct NSEs. This not only allows for the generation of more NSEs, but also creates the opportunity for the inhibition from one NSE to alter the spike timing of the other NDEP, as demonstrated in Figure 7.

Networks of neurons may communicate through changes in firing rate and synchrony; however, an important component of both types of communication is the pattern of this synchronized firing activity. The neurons that convey this information are the excitatory, or projection, neurons, whose axons innervate many networks. The activity of the excitatory neurons is modulated by the inhibition generated by each NSE, allowing for a variety of network firing patterns in response to the timing of the NSEs. Due to the competition between the NDEPs in the network containing two NDEPs receiving DIAF input, we observe a firing pattern that is much more variable than a network containing just one NDEP (see raster plots in Figure 7A for example). With more variability in the firing pattern of the network output, more information (more patterns of activity) can be transmitted to downstream areas.

**Figure 9:**
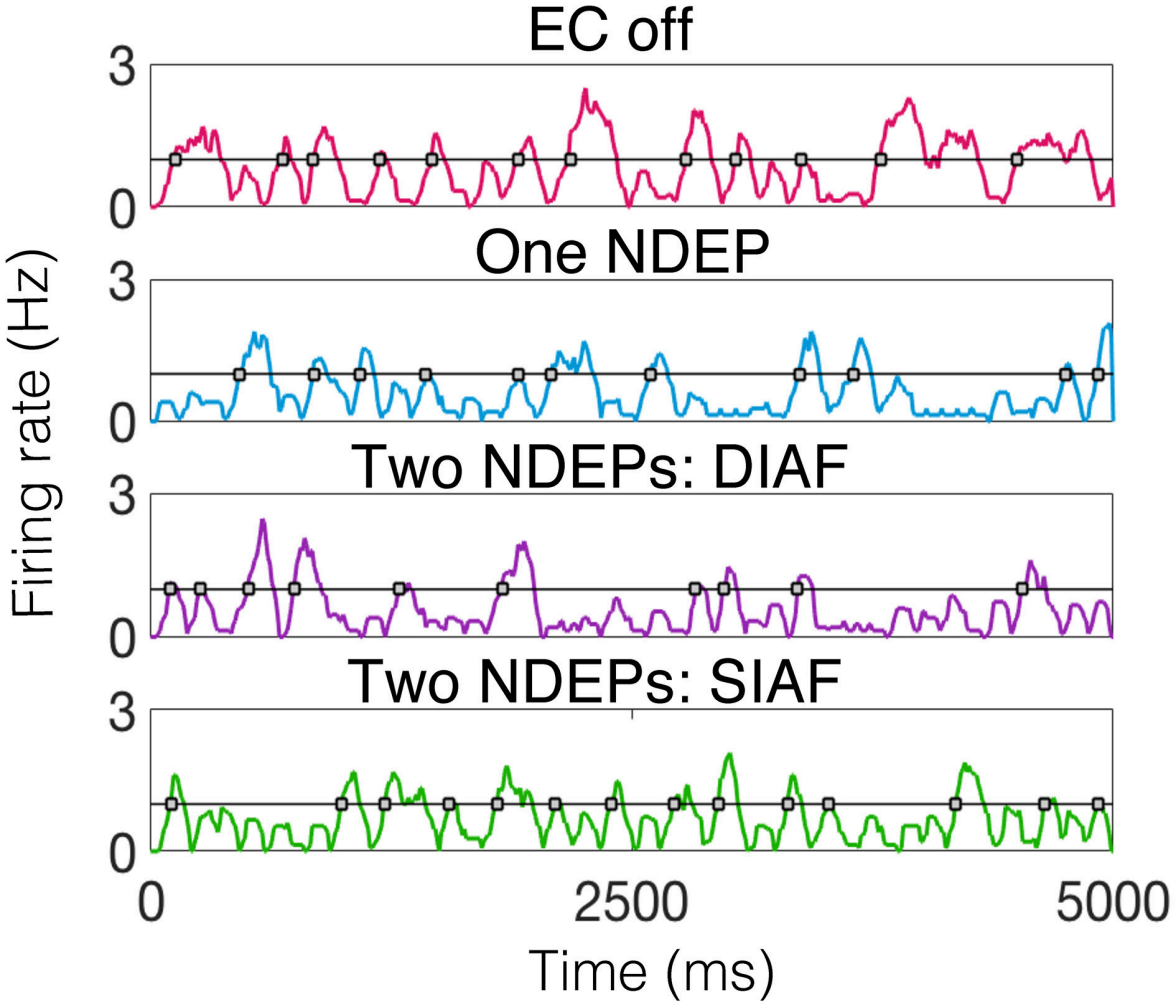
Smoothed instantaneous firing rate of the excitatory neurons in an example of each type of downstream network with the threshold for determining an excitatory event drawn as a black line and times at which excitatory events occurs indicated by gray-filled squares. The input IAF networks were driven with the following parameters for each regime: ν = 5, 000 Hz for EC off and one NDEP, ν = 4, 000 Hz for both two-NDEP networks.

To begin to quantify this variability, we examine the behavior of the excitatory-neuron population by looking at times at which a majority of the excitatory population is active, or the times at which the smoothed instantaneous firing rate (see Input Synchrony in Methods section for the algorithm to compute a smoothed instantaneous firing rate) of the excitatory population crosses a threshold of 1 Hz. For an example of the behavior of the excitatory neurons, for each regime, for one realization, see Figure 9.

**Figure 10:**
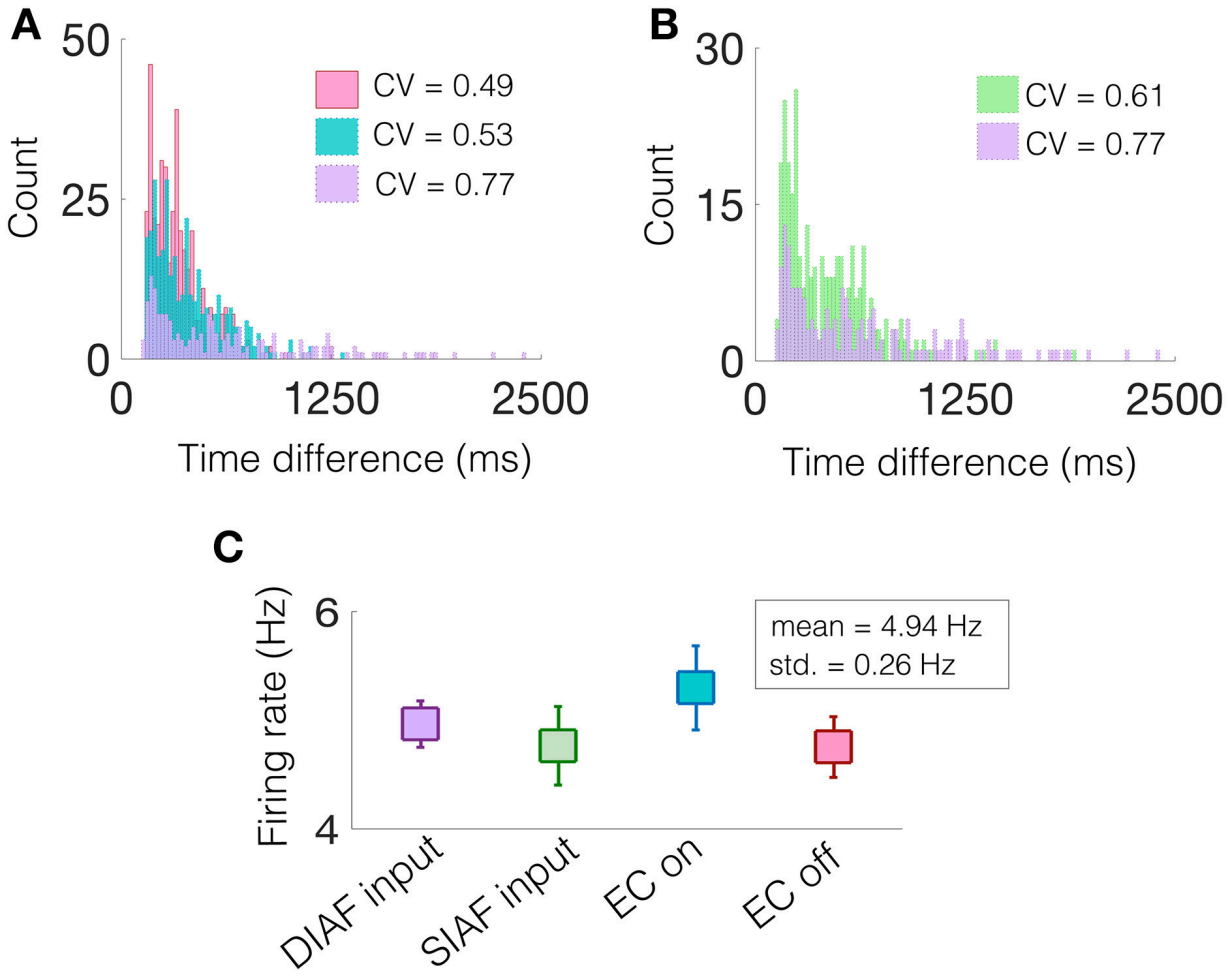
A network containing two NDEPs has greater variability in its firing pattern. **(A)** A histogram of the time difference between excitatory events, or inter-event interval, with EC turned off (pink), one NDEP (blue), and two NDEPs receiving DIAF input (purple). **(B)** A histogram of the time difference between excitatory events comparing the networks in which two NDEPs receive SIAF input (green) and DIAF input (purple). The coefficient of variation (CV), shown in the legend, is computed as the ratio of the standard deviation to the mean of the inter-event interval data. **(C)** The mean firing rate of each network averaged over 30 realizations (squares) together with the standard deviation (line). These results are for input synchrony values of 35 for both two-NDEP networks and 45 for the one-NDEP and EC-off networks, chosen to maintain a relatively constant firing rate across regimes, as shown in **(C)**.

Specifically, we are interested in the pattern of the timing of each of these excitatory events, see gray filled squares in Figure 9. To observe the variability in the firing pattern, we compute the time difference between successive excitatory events, or inter-event interval, for each regime. These time differences are then binned in a histogram with a bin size of 20 ms, see Figures 10A,B for all regimes.

Notice that the distribution of the inter-event interval for the network containing two NDEPs (purple) is broader than for one (blue) and for the case when EC is turned off (pink), as shown in Figure 10A. The amount of variability in the activity of the network can be quantified using the coefficient of variation (CV), or the ratio of the standard deviation to the mean, with higher values indicating more irregularity in the time differences, see legend of Figure 10A. As expected, the CV for the case with two NDEPs receiving DIAF input is significantly larger than the CV for the other regimes. Note that the inter-event interval data for the two-NDEP case with DIAF input has a higher CV value (more variability) than the network receiving SIAF input due to the competition between the NDEPs (see Figure 10B), as discussed previously.

To confirm that the difference in the CV of the inter-event interval is not simply an effect of difference in firing rates, we show that the average firing rate of the neurons in each regime are similar, see Figure 10C. It is clear from the broad inter-event interval histogram, as well as the high CV value, that a network containing two NDEPs has the ability to output a wide variety of firing patterns, leading to a network that has the capacity to code for many different stimuli.

## 4. Discussion

We have developed a model patch of a cortical network, organized in a way that elucidates a possible biological function for a rare number of electrotonic pairs, whose properties agree with experimental results. Measurements of EC between pairs of pyramidal cells in the adult cortex have been elusive to many experimentalists, having only recently been detected and their properties measured (Wang et al., 2010). Further investigations of our work (Crodelle et al., Manuscript in preparation) show that under normal network conditions, i.e., no strengthened synapses, a network with a rare number of electrotonic pairs is practically indistinguishable from one without; the observed effects are local and small, on the order of magnitude of standard network fluctuations. The rarity of these proposed ECs suggests that their role might be in a more specialized network, one in which the connection to the gap-junction-connected interneuron population is enhanced. We have used such a network structure to show that EC between pairs of pyramidal cells could enhance the encoding of external stimuli. Specifically, we are interested in understanding how the NDEP (with EC turned on and off) alters the cortical network response to input that varies from unsynchronized to synchronized, with additional attention paid to analyzing how the varied HH-network output may be interpreted by further downstream areas.

In a network where synchrony is dominated by the gap-junction-connected interneuron population, we have shown that the addition of one NDEP generates many network-wide synchronous events by exciting the interneuron population with tightly synchronized excitatory spikes. These NSEs produce waves of strong inhibition, suppressing the activity of the network and keeping the firing rate of the network relatively low (sparse coding), while the tight synchrony of the NDEP keeps the timing of the NSEs very precise (spike-timing coding). This contrasts the network in which EC is turned off and an NSE can be induced only when a large percentage of excitatory neurons in the network are activated, resulting in high network firing rates and imprecise spike timing.

Coincidence detection in the cortex is an important property of pyramidal cells, in that it enables an efficient response to synchronized sensory input and organizes network activity (Azouz and Gray, 2000; Spruston, 2008; Shai et al., 2015). The tightly synchronized events elicited by the NDEP with EC turned on results in a network that sends very precisely-timed spikes to further downstream networks, aiding in coincidence detection capabilities of downstream areas. Our model predicts that networks in which EC between pairs of pyramidal cells is blocked will exhibit fewer network-wide synchronous events and have less precise spike-time correlation between incoming spikes from upstream networks, those elicited in downstream networks. From our preliminary numerical results, we also observe that the NDEP cannot be effectively replaced by just one excitatory neuron. This is because the spiking statistics of a pair of neurons with EC differs from those without EC in that pair of neurons, i.e., the neuron pair with EC elicits more regularly-timed spikes (lower CV values) and higher firing rates (smaller interspike intervals), when compared to that without EC.

In addition, studies have shown that the activity of interconnected inhibitory interneurons can modulate and influence the activity of the projection cells (Traub et al., 2001; Hjorth et al., 2009). Our investigation of a network with an additional NDEP shows that the two electrotonic pairs can elicit NSEs at their respective spike times, with a non-trivial interaction between the spike timing of each NDEP through inhibition from the interneuron population. The resulting network shows greater coding capacity than a network containing just one NDEP, as well as a network with EC turned off. Though we have only tested two NDEPs in this small-network case, our results predict that the coding capability for many NDEPs might be yet greater. The NSEs elicited by many NDEPs may create even more interactions between the spike timing of the NDEPs, yielding a larger variety of network firing patterns and further increasing the coding ability of the network. Although the size of our model network considered here is relatively small, the obtained results about the role for pair-wise EC in transmitting information across cortex through interactions with the vast gap-junction coupled interneuron populations are expected to extend to large-size neuronal networks.

## Data Availability

All datasets analyzed for this study are included in the manuscript and/or the supplementary files. Computer codes and raw data are available upon request.

## Author Contributions

DC and GK contributed conception and design of the project. JC wrote the code, ran the simulations, and analyzed the results. DZ contributed to the interpretation of the results and generating new ideas. JC wrote the first draft of the manuscript. DZ, GK, and JC contributed to manuscript revision, read, and approved the submitted version.

### Conflict of Interest Statement

The authors declare that the research was conducted in the absence of any commercial or financial relationships that could be construed as a potential conflict of interest.
